# Gut-Brain cross talk: The pathogenesis of neurodevelopmental impairment in necrotizing enterocolitis

**DOI:** 10.3389/fped.2023.1104682

**Published:** 2023-02-15

**Authors:** Krishna Manohar, Fikir M. Mesfin, Jianyun Liu, W. Christopher Shelley, John P. Brokaw, Troy A. Markel

**Affiliations:** ^1^ Department of Surgery, Indiana University School of Medicine (IUSM), Indianapolis, IN, United States; ^2^ Riley Hospital for Children, Indiana University Health, Indianapolis, IN, United States

**Keywords:** gut-brain axis, necrotizing enterocolitis, perinatal brain injury, microbiome, neonatal brain, neurodevelopmental impairment

## Abstract

Necrotizing enterocolitis (NEC) is a devastating condition of multi-factorial origin that affects the intestine of premature infants and results in high morbidity and mortality. Infants that survive contend with several long-term sequelae including neurodevelopmental impairment (NDI)—which encompasses cognitive and psychosocial deficits as well as motor, vision, and hearing impairment. Alterations in the gut-brain axis (GBA) homeostasis have been implicated in the pathogenesis of NEC and the development of NDI. The crosstalk along the GBA suggests that microbial dysbiosis and subsequent bowel injury can initiate systemic inflammation which is followed by pathogenic signaling cascades with multiple pathways that ultimately lead to the brain. These signals reach the brain and activate an inflammatory cascade in the brain resulting in white matter injury, impaired myelination, delayed head growth, and eventual downstream NDI. The purpose of this review is to summarize the NDI seen in NEC, discuss what is known about the GBA, explore the relationship between the GBA and perinatal brain injury in the setting of NEC, and finally, highlight the existing research into possible therapies to help prevent these deleterious outcomes.

## 1. Introduction

Necrotizing enterocolitis (NEC) is a devastating condition that primarily affects premature neonates and is associated with high morbidity and mortality rates (1). The pathophysiology of this disease is multifactorial and is thought to be driven by an immature intestine and immune system, microbial dysbiosis, and a cascade of inflammatory responses (1, 2) that can result in intestinal injury and necrosis, which often progress to requiring surgery and intestinal resection (2). If these neonates survive, they are faced with several downstream complications including intestinal malabsorption, chronic lung disease, and neurodevelopmental impairment (NDI) (3, 4)—much of which is mediated by the complex interplay of the gut-brain axis (GBA). Although it is understood that infants with a history of NEC go on to have worse neurodevelopmental outcomes (3), the pathogenesis of perinatal brain injury in NEC, the causes of downstream development of NDI, and the role of the GBA are not well understood.

The gut-brain axis (GBA) is defined as the interaction of several systems including: the central nervous system (CNS); the autonomic nervous system (ANS); the microbiome; and the many neural, immune, and hormonal signaling pathways that exist between them (5–9). Alterations in the neonatal microbiome and the intestinal injury seen in NEC contribute to pathogenic alterations in GBA signaling (5). This activation then triggers the downstream CNS inflammatory cascade seen in perinatal brain injury, which involves the activation of microglia—the main mediator of the innate immune system's response to brain injury (10). In addition, the stress of prematurity, maternal separation, and formula feeding can further activate the GBA in reverse and exacerbate this disease process (9). NEC usually occurs during a period of crucial and dynamic neurological development leaving the infant particularly susceptible to the pathogenesis of this disease (11), which leads to both short and long-term neurodevelopmental impairment (12). The purpose of this review is to summarize what is known about neurodevelopmental outcomes in NEC, the proposed interplay of the gut-brain axis in the pathophysiology of this disease, and to highlight research into possible therapies to help improve these detrimental outcomes.

## 2. Neurological and developmental delay seen in NEC

The presence of neurological changes and subsequent NDI in patients with a history of NEC is well established (13, 14). The systemic inflammatory response triggered by NEC may be mediated *via* bacterial products and cytokines released during intestinal injury. This, combined with the associated hypotension that is part of the systemic inflammatory response, results in signals traversing the GBA and causes well-documented white-matter injury (6, 15). Other neural changes noted include alterations of the brain parenchyma, decreased head circumference, and corresponding decreased volumes in total brain matter (16, 17). This stunted head growth and altered brain parenchyma in early infancy are detrimental to later cognitive outcomes and result in downstream NDI including: a higher incidence of cerebral palsy(CP), impaired motor function, visual and hearing impairment, and cognitive deficits (14, 16, 17).

### 2.1. Assessing neurodevelopmental impairment

There are a barrage of developmental screening tests for children used for early detection of developmental delays with the goal of identifying if a child has reached specific physical, cognitive, social-emotional, and behavioral milestones (18). It is important to keep in mind that these milestones are often modified by historical and cultural factors and the assessments themselves are limited by the training, availability of assessors, and the education level and socioeconomic status of parents. Despite these difficulties, there remain a series of established assessments that aim to evaluate these milestones, however, no established assessment and timing of assessments exist for looking at NDI in NEC. For this review, we will focus on describing a few assessments that are specifically targeted and validated for screening for developmental delays in high-risk populations (19, 20).

#### 2.1.1. Ages and Stages Questionnaire -3rd edition (ASQ-3)

The ASQ-3 is a developmental screening tool that utilizes a parent-centric model. This questionnaire can be used in both general primary care and in higher-risk categories such as evaluating children that were born prematurely. The questionnaire is given at pre-determined ages (adjusted for corrected gestational age) and tracks the developmental progress of children between the ages of one month to just over 5 years. The benefit of this questionnaire is that it has an easy learning curve for administration, has several different language options, and is quick to administer (20, 21).

#### 2.1.2. Bayley scales of infant and toddler development

The Bayley Scales of Infant and Toddler Developmental assessment is a widely used and the most psychometrically sophisticated assessment of development in infants and toddlers. This scale is advantageous because it is especially useful to screen high-risk populations such as those infants that are pre-term, have lower birth weight, or are from a lower socioeconomic status. It assesses cognition, language, motor, social-emotional, and adaptive behavior with an administration time ranging from 30 to 90 min. Most studies looking at NDI use this assessment, however, the drawbacks are that it is a difficult assessment to administer—requiring specialty training and a lengthy period of time with the patient and their families (19, 20).

#### 2.1.3. Cognitive adaptive test/clinical linguistic auditory milestone scale (CAT/CLAMS) (20)

Like the Bayley Scales Assessment, the Cognitive Adaptive Test/Clinical Linguistic Auditory Milestone Scale (CAT/CLAMS) is another assessment that is practitioner-administered and specifically advantageous for high-risk children—especially from pre-term or low-birth-weight populations. It is a relatively newer assessment but compares favorably to the Bayley assessments and looks at language, problem-solving, and visual motor skills in children from birth to 3 years old. The CAT/CLAMS is also especially useful because it has high validity to target and identify early language delays (20, 22).

### 2.2. Clinical changes and neurodevelopmental impairment in NEC

A systematic review performed by Rees et al. in 2006, looked at 7,843 premature children (821 of which had NEC) and their neurodevelopmental milestones over an average of 20 months. These results demonstrated that infants with a history of NEC were more likely to have some form of neurodevelopmental impairment (NDI). Specifically, the breakdown showed that 20% of the patients with NEC developed CP, 3% developed visual impairments, 3% hearing deficits, 36% cognitive deficits, and 35% psychomotor impairments. Interestingly, when the data was further stratified, those with medical NEC were not found to have significant neurodevelopmental impairment when compared to the cohort without NEC (prematurity alone), while those in the “surgical NEC group” had a more significant impairment, worse outcomes, and higher rates of CP and psychomotor impairment overall (12). Another review analyzed a database of 12,992 very low birth weight (VLBW) infants in Israel and looked at the association of several neonatal co-morbidities (including NEC) with the risk of head growth failure (HGF)—defined as head circumference z-score that was greater than two z-scores below the mean. Overall, the risk of severe HGF was associated with a nearly 3-fold greater odds with a diagnosis of NEC. These differences are even more disparate when surgery becomes necessary, and infants diagnosed with surgical NEC had an odds ratio of 7.62 associated with the development of severe HGF (23).

The pathogenesis of NEC progresses to requiring surgery for several reasons including, free intra-abdominal air and/or clinical deterioration despite optimal medical management—often translating to worse outcomes in infants with “surgical NEC” (24). The disparity between NDI in medical and surgical NEC is further illustrated by a multi-center retrospective review of 2,948 extremely low birth weight (ELBW) infants. At a corrected age of 18 to 22 months, infants with “surgical NEC” were found to have significantly reduced weight, length, and head circumference when compared to infants without NEC or with medical NEC. On Bayley Scales of Infant Development assessments, surgical NEC, but not medical NEC, was found to be an independent risk factor for lower scores on the mental developmental index (MDI), psychomotor developmental index (PDI), and resulted in an increased risk of neuro-developmental impairment (NDI) (25). This disparity in surgical vs. medical NEC is highlighted again by a study by Martin et al. looking at a cohort of 1,155 neonates for the development of surgical or medical NEC and accompanied prognostic factors. Those who had both surgical NEC and late bacteremia had worse NDI, with the group citing an increased risk of CP [OR = 8.4 (1.9, 39)] and microcephaly [OR = 9.3 (2.2, 40)]. Like the previous study, children with medical NEC with or without late bacteremia were not at increased risk of any developmental dysfunction (26).

It is also important to note that NDI seen in early childhood testing often persists in school-aged children. Rose et al. looked at neurodevelopmental outcomes of school-aged children with a history of surgical NEC or SIP (spontaneous intestinal perforation) and compared them with matched controls (27). Although this study combined outcomes from SIP and NEC, the data still showed that the combined cohort had more abnormal motor function scores (as assessed by Movement Assessment Battery for Children) and lower intelligence quotients (IQ)-(86 ± 14 compared with 97 ± 9 in the controls) (27), supporting the hypothesis that NDI persists past infancy.

### 2.3. Necrotizing enterocolitis (NEC) and spontaneous intestinal perforation (SIP)

Spontaneous intestinal perforation (SIP) is a discrete entity from NEC and is characterized by an isolated perforation in the gastrointestinal tract. The presentation of an infant with SIP and NEC with perforation can be similar, however, the significant systemic inflammatory response of NEC isn't seen in SIP patients with these infants faring better after resection (28). Although NEC and SIP are often grouped together, NEC has been shown to have more significant NDI as evidenced by a retrospective study on preterm infants that compared neurodevelopmental outcomes within a cohort of NEC and SIP patients (29). A battery of neurodevelopmental assessments showed more significant abnormal findings in NEC compared to SIP in gross and fine motor skills as well as cognitive deficits (29), suggesting that the inflammatory process of NEC plays a greater role in brain injury and development of NDI.

The severity of NEC and the need for surgery demonstrating worse NDI lends itself to the question if surgery itself contributes to the NDI seen. The Necrotizing Enterocolitis Surgery Trial (NEST) looked at 310 extremely low birth weight infants (EBLW) and evaluated the difference between initial laparotomy vs. drainage on the rates of death or NDI (data collected from 18 to 22 months) in NEC and SIP. NEST ultimately determined that initial laparotomy was more likely to reduce rates of death or NDI in infants with a preoperative diagnosis of NEC when compared to placing a Penrose drain (30). This data echoes an earlier observational study in 2006 that showed that NEC (when compared to SIP) had a higher-odds of death and NDI at 18–22 months of adjusted age (31). These data indicate that surgical intervention itself, is not the primary driver of NDI, as those with worsening clinical NEC did better with more aggressive surgical intervention(laparotomy) vs. leaving a drain in place.

The above studies demonstrate that surgical NEC has worse NDI outcomes than SIP, and it is the progression of NEC pathogenesis to requiring surgery that leads to worse NDI (not surgical intervention alone). A retrospective analysis of preterm infants by Bell et al. clarifies this issue further and looks at outcomes of patients with NEC and SIP with or without short bowel syndrome (SBS). The risk of development of moderate to severe NDI was 77% in the cohort of infants with NEC/SIP and SBS when compared to 62% of those with NEC/SIP without SBS (aRR 1.22) and 44% with no NEC, SIP, or SBS (aRR 1.6). In addition, children developing short bowel syndrome had lower cognitive, language, and motor scores on Bayley assessments than the cohort with NEC/SIP that didn't develop SBS (32). Although this study didn't differentiate between surgical NEC and SIP, it did highlight that the surgical resection of intestine—resulting in short-bowel syndrome (SBS)—is another contributing factor to the development of long-term NDI. In summary, the existing literature on NDI indicates that surgical NEC has high rates of NDI when compared to SIP, medical NEC, and prematurity alone. In addition, the development of SBS results in even higher NDI.

### 2.4. MRI changes in parenchyma correspond to NEC severity

The presence of increased parenchymal abnormalities in NEC patients as seen on magnetic resonance imaging (MRI) has been validated in several studies. MRIs performed on a prospective cohort of 192 premature infants at birth and repeated at 2 years old showed that infants with sepsis and/or NEC had a higher prevalence and severity of white matter abnormality, and specifically that infants with NEC had higher rates of concurrent gray matter abnormality. Unsurprisingly, infants with surgical NEC had more severe brain injury detected on MRI when compared with infants with medical NEC. When adjusted for other factors, this cohort was also found to have delayed cognitive and motor impairment (17) as demonstrated in the studies described earlier. In another study of 26 premature infants with NEC or SIP, infants with surgical NEC and SIP were found to have more significant brain injury seen on MRI, when compared with infants with medical NEC, even after adjustment for confounders (33). It is important to note that the patients with SIP were combined in the group with surgical NEC, so we are unable to extrapolate about the difference in significance between SIP and surgical NEC brain injury on MRI. More recently, another study looked at 69 infants with surgical NEC and found that 52% had some form of white matter brain injury as seen on MRI and were subsequently more likely to have a severe postoperative course. Those that survived with known white matter brain injury were found to have lower mean motor, cognitive, and language scores as well as higher rates of visual impairment at 2 years of age (34). These studies together show that NEC severity corresponds to parenchymal changes and especially white matter injury as seen on MRI. These observations along with others (17, 34) support the hypothesis that bowel injury initiates inflammation that potentially affects the developing brain (26).

## 3. The Gut-Brain Axis: an explanation for neurodevelopmental impairment

The gut-brain axis (GBA) is a bi-directional highway of communication involving neuro-immune-endocrine mediators that link the gut, the microbiome, and the nervous system—playing a critical role in the homeostatic processes of health and disease (9, 35). The alteration of the GBA has served as a framework for the explanation of many diseases for over three decades (36) and is now acknowledged as a crucial part of the development of the pathogenesis and downstream NDI in NEC (36, 37).

**Figure 1:**
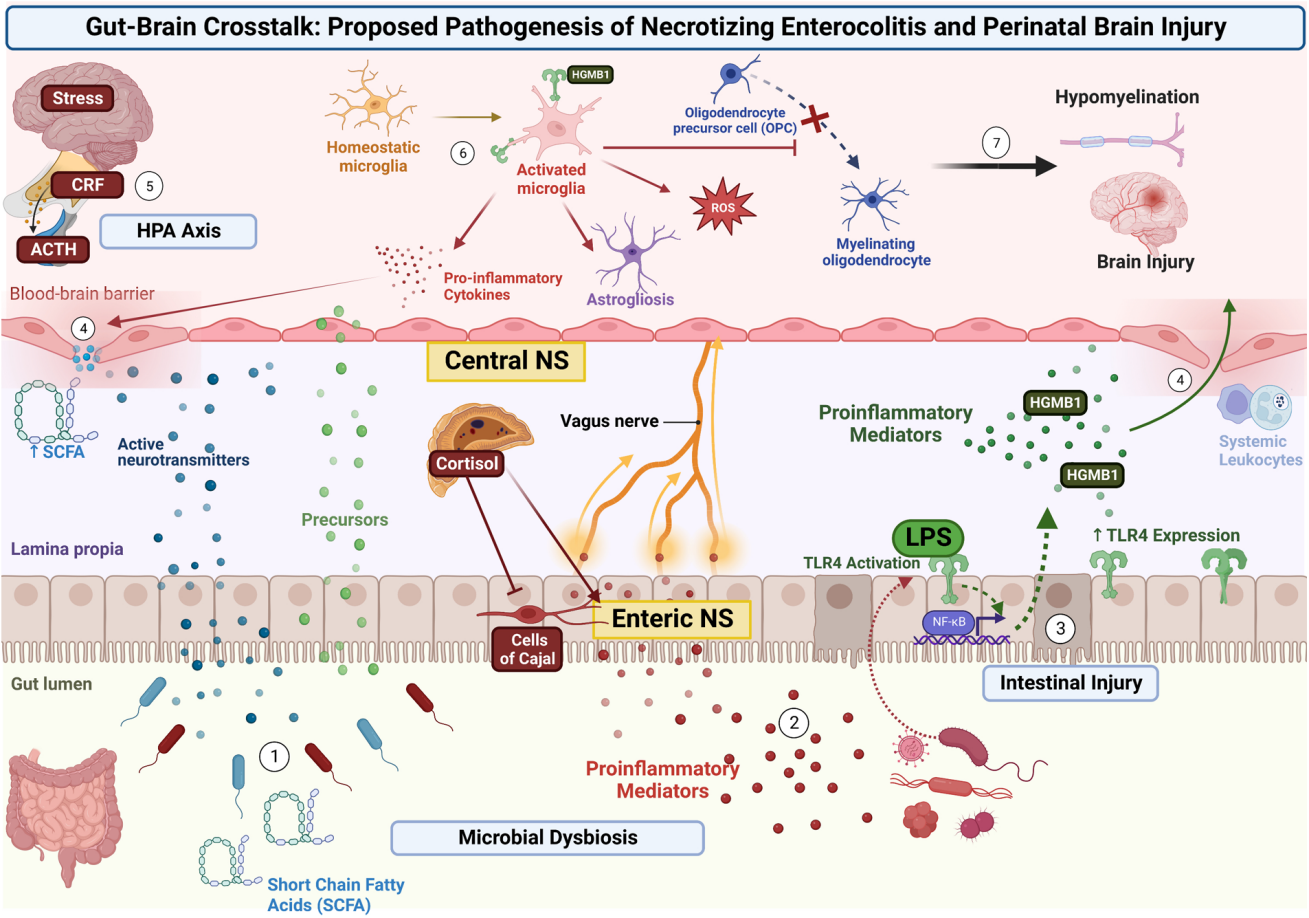
Proposed pathogenesis of NDI in NEC *via* the Gut-Brain Axis. Microbial dysbiosis and subsequent intestinal injury leads to the activation of several signaling pathways. (1) Pathogenic bacteria release signaling molecules including neurotransmitters, gasotransmitters, and short-chain fatty acids that cross the intestinal membrane and enter the systemic circulation. (2) Pathogenic bacteria release proinflammatory cytokines and other inflammatory mediators to stimulate the ENS within the intestinal wall, with signals traveling through the Vagus nerve, and ultimately to the CNS. (3) Intestinal injury and release of mediators (such as LPS) results in TLR-4 signaling and increased expression. This results in transcriptional changes *via* NF-κB and further release of inflammatory mediators into the systemic circulation. (4) Systemic and neural inflammatory mediators weaken the BBB and allow further passage of pro-inflammatory mediators and systemic leukocytes. (5) Concurrently, stress is processed in the limbic system of the brain, resulting in release of CRF from the hypothalamus, release of ACTH from the pituitary, and the release of cortisol from the adrenal gland. This signaling through the HPA axis and increase in systemic cortisol results in further activation of the ENS and intestinal epithelial cells, while inhibiting interstitial cells of Cajal (resulting in decreased gut motility). (6) Signals and inflammatory mediators reach the CNS and cause local brain injury as well as activate microglia *via* ligands (such as HMGB-1) binding to TLR4. Activated microglia release pro-inflammatory cytokines and free radicals, stimulate astrocytes, and injure developing pre-oligodendrocytes. (7) The combination of these insults results in brain injury and hypomyelination.

In the case of NEC, this begins as a combination of microbial dysbiosis and subsequent intestinal injury. This leads to signals traveling *via* the enteric nervous system (ENS) (6) residing within the intestinal wall, through the vagus nerve, and ultimately to the central nervous system (CNS) (6, 7). In addition to neural signaling, pathogenic bacteria release lipopolysaccharide (LPS) and a variety of other inflammatory mediators (such as fatty acids) into the systemic circulation. This initiates a cascade of inflammatory factors that causes systemic inflammation but also activates toll-like receptors on microglia. Activated microglia release pro-inflammatory cytokines, free-radicals, and help to activate other cells such as astrocytes as well as injure developing pre-oligodendrocytes. The combination of these insults results in white matter injury (7, 38). The interplay of this axis and its suspected role in NEC is further detailed in the following sections and is illustrated in Figure 1.

### 3.1. The role of the microbiome

The microbiome is a critical part of the GBA with microbiota influencing the CNS by interacting locally with intestinal cells and the ENS or directly *via* neuroendocrine and metabolic mediators (8, 9). The importance of the microbiome to the homeostasis of the GBA is best highlighted by the wealth of studies of germ-free animals which have shown a wide array of impairment or dysregulation in immune function, amino acid metabolism, hormone signaling, neurotransmission, and behavioral phenotypes when compared to their counterparts (9, 39–42).

Dysbiosis—or the change of the microbiome towards an unfavorable or pathogenic bacterial colonization—is a major contributing factor to the development of NEC (36, 43, 44). The infant microbiome is normally characterized by large amounts of *Lactobacillus*, *Bifidobacterium*, and *Bacteroides* in the first few weeks to one year of life which is aided by normal vaginal delivery and feeding with human breast milk (45). Although there are no causal species to the development of NEC, an overgrowth of gram-negative organisms, specifically in the *Enterobacteriaceae* family (46, 47) and loss of intestinal diversity can contribute to dysbiosis and the multifactorial etiology of NEC (44, 47). Additionally, several experimental models have shown the pivotal role of bacteria in the pathogenesis of NEC as germ-free animals are protected from developing NEC (46, 48).

This unfavorable change in the microbiome triggers an acute inflammatory response that leads to further disruption of the already immature intestinal barrier and is further exacerbated when pathogenic bacteria release their endotoxins and pro-inflammatory mediators or translocate across the intestinal mucosa (49). The microbiome also directly influences the brain microenvironment by the generation of neurotransmitters, short chain fatty acids (SCFAs), and cytokines as well as *via* direct activation of immune cells and communication with neural networks that traverse up to the CNS (35, 50, 51). Bacterial toxins such as Lipopolysaccharide (LPS) can reduce ENS activity and inhibit the function of interstitial Cells of Cajal—often referred to as the pacemaker of the intestine and important to gut motility—resulting in the ileus that is often seen in NEC (36, 52). The microbiome also is an important regulator of the hypothalamic-pituitary axis (HPA) and is important for the postnatal development of an appropriate HPA stress response in mice. Activation of this axis can result in elevated levels of systemic cortisol which can further cause intestinal injury and stimulation of the pro-inflammatory cascade (8, 42). The microbiome also plays a role in the regulation of important epithelial barriers. Changes in the microbiome can cause direct influences on the intestinal epithelium and tight junction barrier activity (8). Additionally, studies in germ-free mice have shown that a healthy microbiome is essential to the development and function of the blood brain barrier (BBB), with germ-free mice showing increased BBB permeability that persisted into adulthood. In these studies, restoration of BB integrity was seen by postnatal recolonization of the intestine with a probiotic (9, 53).

#### 3.1.1. Toll-Like receptor signaling

Toll-Like Receptors (TLRs) are pathogen-associated molecular pattern recognition receptors that participate in signaling in response to infection or disease (36). TLR-4 signaling, specifically, plays a pivotal role in the GBA and the pathogenesis of NEC. It has been shown that TLR-4 activation is unregulated in preterm infants and that TLR-4 knockout animals do not develop NEC (48, 54). Lipopolysaccharide (LPS), an endotoxin produced by pathogenic bacteria, results in an excessive TLR-4 activation in intestinal cells that causes translocation of transcription factors such as nuclear factor kappa-*β* (NF-kB) leading to the transcription of various pro-inflammatory cytokines and other mediators (48, 55). These mediators cause intestinal inflammation, disrupt mucosal integrity, and enter the systemic circulation. From here, these mediators initiate a systemic inflammatory response and can travel through a weakened BBB to further initiate damage and activate the brain's immune response (48, 51, 54–56).

#### 3.1.2. Short Chain Fatty Acids(SCFAs)/neurotransmitters/gasotransmitters

Gut bacteria also independently produce metabolites that participate in the GBA. Both commensal and pathogenic bacteria produce SCFAs, such as butyrate, propionate, and acetate, that play a role in maintaining the barrier function of intestinal epithelial cells. Butyrate specifically can serve as a fuel source for colonocytes and improve tight junction integrity (35, 57). Alteration in the balance of gut SCFA is implicated in the altered function of both the intestinal barrier and BBB as well as the maintenance of homeostasis in the CNS (9). These molecules are also known to stimulate the sympathetic nervous system and influence the memory and learning process (8). SCFAs can diffuse through epithelia to exert their effects, typically through the inhibition of histone deacetylase (9, 58). In mice, intraperitoneal injections of butyrate have been shown to enhance learning, memory, and sociable behaviors while simultaneously decreasing depressive-like behaviors (9, 59–61). Studies in animal models have also shown that SCFAs can induce vagus nerve activation (9) and enhance mucosal barrier protection (9, 62). Although many studies promote the benefits of SCFA, the alteration of the homeostasis of SCFA has been implicated in certain disease processes (9). Specifically, excessive production of SCFAs has been implicated in NEC. One possible explanation for this can be secondary to overproduction of SCFAs by bacteria and poor gut motility which can in turn cause local accumulation of SCFAs (56, 63).

In addition, bacteria can both directly release neurotransmitters (such as 5-HT and GABA) (8, 9), molecules that mimic local neurotransmitters (8), and can stimulate intestinal cells to release neurotransmitters, which traverse the intestinal epithelium. These molecules or their precursors can then pass through a weakened BBB and further influence the brain (64) and CNS physiology, resulting in possible brain injury and altered development (11, 65). Gasotransmitters are another important type of signaling molecule in the GBA. In NEC, an emerging gasotransmitter of interest is H_2_S. Commensal bacteria such as *Lactobacilli* can produce hydrogen sulfide which further modulates gut motility (66). In fact, H_2_S has been shown to have protective effects on intestinal injury in murine models of NEC (8, 9). Although the effect of H_2_S on NDI has not been illustrated, there have been studies on neuroprotective effects of H_2_S in secondary brain injury after a TBI (67). In rats, intraperitoneal injection of NaHS, a H_2_S salt, resulted in improvement in TBI-induced memory impairment (67, 68) and H_2_S decreased TBI induced lesion volume in brains (67, 69).

### 3.2. Neural communications and the Hypothalamic-Pituitary Axis (HPA)

There are several neural pathways that allow the peripheral components of the GBA to communicate with the brain (36). The ANS afferent pathway starts with signaling from the lumen which traverse through the enteric nervous system (ENS) and vagal nerve to reach the CNS (8). The efferent pathway (from the CNS back to the intestinal wall) (8) often serves an anti-inflammatory function. In healthy individuals, this pathway helps to balance out or “check” the responses secondary to pro-inflammatory cytokines such as TNF-α (tumor necrosis factor alpha), signaling molecules such as HMGB1 (high mobility group box1), and inflammasomes (multi protein cytoplasmic complex that triggers cascades to enhance secretion of proinflammatory cytokines)—thereby preventing unregulated pathogenic signaling (36).

The ENS is the first access point to the afferent pathway and resides within the intestinal wall (36)—receiving signals from microbiota, immune cells in the epithelium, and altered and injured intestinal epithelium (36). Enteric signals can then communicate through the vagus nerve, dorsal root, and nodose ganglia to the CNS (6, 35, 36). The vagus nerve serves as a major pathway between microbial mediators, the ENS, and the brain, which is well supported by animal models that show the absence of neurochemical and behavioral effects with the alteration of the microbiome in vagotomized animals when compared to controls (8).

The hypothalamic-pituitary axis(HPA) is a hormonal mediator in the GBA and works alongside other signaling pathways (36). The microbiome also is a regulator of the HPA and has shown to be important to for the postnatal development of an appropriate HPA stress response in mice (8, 42). If the gut is “stressed” or there is dysbiosis, the HPA processes this information up in the limbic system. This results in corticotropin-releasing factor (CRF) from the hypothalamus, adreno-corticoid hormone(ACTH) secretion from the pituitary (8, 9) and ultimately stimulates the adrenal gland to release cortisol (9). The activation of this system then allows neural-hormonal influence of immune and intestinal epithelial cells, interstitial cells of Cajal, and ENS neurons (8, 36). Stress and signaling from the brain can drastically affect the intestine by alteration of intestinal permeability (8) and immune cell activation (8).

### 3.3. The brain: immune cell signaling and the brain's effector cells

The brain itself, is a complex signaling system of regions that have their own sensory and motor functions and includes the cerebral cortex, the cerebellum, the limbic system, the HPA, and the brain stem (36). Injury to different parts of the brain, can have several downstream consequences to immune functioning, neurobehavioral disorders, and intestinal disease processes (36). For the purposes of this review, we are specifically interested in neural damage as signals travel up to the brain from injury in the intestine as in the case of NEC.

Once pathogenic signals have travelled through the vagus nerve and/or inflammatory mediators have reached the CNS, the brain becomes especially susceptible to injury. This process includes activation of microglia (*via* TLR4 stimulation) and subsequently astrocytes and glial cells within the brain (70). Activated microglia and astrocytes migrate to sites of injury and begin the neuroinflammatory cascade by releasing pro-inflammatory cytokines such as TNF-α, interleukin-1β (IL-1β), and interleukin-6 (IL-6) (70–73). The BBB (already weakened by SCFA release) is further destabilized by the release of cytokines and the inflammatory activation of enzymes such as matrix metallopeptidases (MMPs), which allows systemic leukocytes to enter and further exacerbate injury. This interplay is believed to cause abnormalities in normal myelination and white matter injury (70, 73). At the cellular level, white matter injury is defined by alterations in the developing oligodendrocytes and specifically the pre-myelinating oligodendrocyte cell which results in hypomyelination (38, 73). Understanding these cellular and molecular processes is important for identifying future targets for prevention of poor neurodevelopmental outcomes after NEC.

## 4. Animal studies of NEC and neurological impairment

Although the clinical and macroscopic neurological effects of NEC on infants are clear, the microscopic changes caused by NEC in the GBA remain to be elucidated. Several animal models of NEC have been studied that show early progress in this realm (13). In mice, Sampah et al. and Nino et al. showed the onset of NEC in the intestine leads to excessive TLR4 signaling and activation of an endogenous ligand HGMB1 (high mobility group box 1) which enters the systemic circulation and activates TLR4 receptors on microglia in the brain. The microglial activation and damage in the brain was confirmed by either an increase in Iba-1(a microglial marker) staining, increased radical oxygen species accumulation, or reduced myelin basic protein (51, 54). Nino et al. further demonstrated that mice exposed to NEC had severe deficits in spatial working memory and novel object recognition memory by the time they reached postnatal day 60 (54). In another murine model of NEC, Biouss et al., showed that pups with NEC had higher brain-to-body weight ratios, thinner cortices, and increased levels of apoptosis and endoplasmic reticulum stress compared to breast-fed controls. In addition, the brains of mice with NEC had an associated reduction in the number of neurons, oligodendrocytes, and neural progenitor cells in specific regions of the brain. Finally, levels of pro-inflammatory cytokines, the density of activated microglia, and the density of astrocytes were increased in the brain, and correlated with an increase in the levels of pro-inflammatory cytokines in the gut and intestinal histologic damage (74).

Other animal models of NEC looking into the GBA remain sparse. A pig model by Brunse et al. showed that preterm pigs undergoing experimental NEC had increased BBB permeability and CNS inflammation (increased IL-6 production), but showed no effect on cerebral myelination or microglia density by day 5 (75). A rat model of NEC showed that animals with NEC demonstrated slower times to reach certain developmental milestones, increased anxiety-like behavior, and decreased cognitive function when compared to breast fed pups. These clinical observations were associated with increased numbers of “activated microglia” and decreased myelin basic protein (76).

The studies taken together highlight a few key findings of the pathophysiology of NEC: 1) intestinal inflammation and injury translates to neural changes that are proposed to occur through TLR4 signaling in the intestine, 2) endogenous ligands released from intestinal TLR4 activation go on to activate TLR4 on microglia and 3) downstream neurologic changes occur including microglial activation, increased neuroinflammation, and decreased myelination which can lead to downstream neurodevelopmental deficits.

## 5. Therapies in perinatal brain injury

The literature centered on therapies to prevent NDI in infants with NEC is scarce, and to date, no strong, randomized clinical trial data exist. However, there are several studies looking into therapies to prevent or ameliorate perinatal brain injury (of which NEC is a known risk factor). The following sections will summarize what is known about therapies to target perinatal brain injury which can be potentially applied to the brain injury seen in NEC.

### 5.1. Stem cell therapy

Mesenchymal stem cells (MSCs) are among the most widely studied stem cells because they are multi-potent cells that are relatively easy to isolate and maintain in culture (77, 78). Furthermore, they have a lower tumorigenic potential and are immune privileged with minimal host immune activation upon administration (79, 80). They have been used in various pre-clinical studies (81–84) and have been shown to reduce inflammation (85, 86), exhibit antioxidant properties (87), enhance neovascularization (88), and improve functional recovery of injured tissues. They can migrate to damaged tissues or organs in response to inflammatory mediators where they act in the local environment *via* secretion of paracrine mediators and interaction with surrounding cells (79, 89). The application of stem cells for the treatment of NEC, is largely still limited to pre-clinical animal studies, and very little is known about the effect of stem cells on NDI (82, 90–94). However, there are some studies that have looked at the separate effects of MSCs on the neonatal diseases of the gut, such as NEC, as well as the effects of MSCs on certain types of perinatal brain injury, including: periventricular leukomalacia (PVL), hypoxic-ischemic encephalopathy (HIE), and neonatal stroke. The combination of these findings helps us to extrapolate the connection between the effect of MSCs on the GBA in the pathogenesis of NEC (79).

#### 5.1.1. MSCs and necrotizing enterocolitis

Over the past decade, stem cells have been studied as a potential avenue of treatment, however the therapeutic benefit of MSCs in the intestinal pathogenesis of NEC has yet to be fully explored in the clinical setting (77, 82, 95, 96). In fact, only one clinical case report shows a benefit of stem cells used in a case of surgical NEC where umbilical-cord-derived-MSCs (UC-MSCs) were given intravenously. Following administration of UC-MSCs, mesenteric doppler imaging showed improved perfusion to prior compromised portions of intestine by post-operative day 4 (97).

There are several animal studies that showcase the benefits of MSCs to mitigate the intestinal pathogenesis of NEC. In rat models of NEC, intraperitoneal(IP) injections of MSCs have shown an improvement in clinical sickness and intestinal histology injury—characterized by restoration of villi-crypt morphology and epithelium along with restoration of populations of Paneth cells, SOX9 cells, and LGR5 stem cells that occupy this crypt niche (98, 99). An adult mouse-model of ischemia and reperfusion utilized several different MSC's including umbilical cord (UC-MSC), bone-marrow (BM-MSC), and adipocyte-derived (AD-MSCs) cells and similarly showed improved overall survival, intestinal perfusion, restoration of normal intestinal histology, and a decrease in pro-inflammatory chemokines (84, 100).

#### 5.1.2. MSCs and perinatal brain injury

Researchers have identified various causes that result in perinatal brain injury including neuronal cell death, ischemia from placental or umbilical cord disruption, accumulation of free radical oxygen species, persistent inflammatory cascades, and defective myelination of neuronal cells largely from microglia-mediated damage of pre-oligodendrocytes (101, 102). No human data exists that looks specifically at the effects of MSCs in neurodevelopmental impairment in NEC, however there are a few animal studies and clinical and preclinical trials that show promise in the field of perinatal brain injury. Oppliger et al. showed that UC-MSCs improved myelination and decreased microgliosis and astrogliosis in a rat model of white matter brain injury (103). A systemic review of 18 murine studies on the effect of neural stem cells (NSCs) on perinatal brain injury showed significantly improved motor function and cognitive function (104) consistently throughout most of the studies. In a preterm sheep model of LPS-induced white matter injury, treatment with UC-MSCs reduced cell apoptosis/inflammation, promoted oligodendrocyte survival,and attenuated astrogliosis (105). Although the overall data behind MSCs in perinatal brain injury, and specifically from inflammatory causes (not ischemia/hemorrhage), is sparse, it shows promising results, indicating that continuing to investigate the benefits of MSCs on improving NDI in NEC would be beneficial. In the following sections, we will delve into specific neonatal brain pathologies and the studies that utilize stem cells to treat them.

##### 5.1.2.1. MSCs in neonatal stroke

The pathogenesis of neonatal stroke involves an ischemia-reperfusion injury with disruption of arterial or major venous flow. Studies in a newborn rat model of neonatal stroke by Kim et al. showed that MSCs reduced brain infarct volume and enhanced astrogliosis and ultimately improved functional test scores (106). Another rat model study of neonatal stroke by van Velthoven et al. showed that intranasally delivered MSCs reduced loss of brain matter and ultimately improved motor function (79, 107).

##### 5.1.2.2. MSCs in Hypoxic-Ischemic Encephalopathy (HIE)

HIE is a perinatal brain injury where insufficient blood flow and oxygen is delivered to brain tissue resulting in damage and disability such as CP. Currently, therapy for HIE centers around hypothermia which prevents secondary brain injury but offers no restorative function (108). There are a few preclinical and animal studies that demonstrate the benefit of MSCs in treating HIE. In a rat model of HIE, the combination of UC-MSCs and hypothermia resulted in a reduction of the previously injured brain region and improved sensorimotor function (109). In addition, MSC therapy showed improvement in the neuro-microenvironment with decreased pro-inflammatory mediators, decreased microgliosis and astrocytosis, and decreased permeability of the BBB (79, 109). Another rat model of HIE demonstrated that intranasally delivered MSCs reduced markers of neuroinflammation and restored neuronal cell numbers (110). In a mice model of HIE, a single MSC infusion treatment directly into the cerebrum resulted in inducible gene expression that promoted growth, proliferation, and survival of neural progenitor and glial cells (107).

Early clinical trials in preterm infants suffering from HIE indicate that autologous UC-MSCs delivered intravenously showed improved Bayley III Assessment scores by 1 year of age (111, 105). UC-MSCs therapy has also been used in older children with CP in which improved cognitive effects and gross motor function have been shown (112, 79, 105). Taken together, these studies show promise in the role of MSCs in neurogenesis and repair (79).

##### 5.1.2.3. MSCs in Periventricular Leukomalacia (PVL)

PVL has a multifactorial etiology including HIE, trauma, immature brain development, and inflammatory changes (79) and is specifically characterized by a loss of pre-oligodendrocytes, loss of normal myelination potential, and diffuse gliosis (79, 113). In a neonatal rat model of PVL, rats receiving intracerebral injections of MSCs demonstrated increased anti-myelin immunoreactivity and glial cell migration and proliferation in injured areas indicating a neuroprotective and neuro-regenerative effect (114). Two different studies of a rat model of PVL illustrated that IP injections of UC-MSCs could replicate this improvement in brain injury with increased mature oligodendrocyte counts, decreased reactive astrocytes, and activated microglia (115) as well as a demonstrated reduction in IL-1B and reversed demyelination (measured by myelin basic protein staining. Interestingly, UC-MSCs pretreated with interferon-gamma resulted in even more significant effects, indicating that MSCs can be primed to deliver their protective effects (79, 116) These studies together show that MSCs delivered in the peritoneum can participate in the GBA to deliver neuro-regenerative effects in the setting of this disease.

### 5.2. Extracellular vesicles (exosomes)

There is a large body of research suggesting that stem cells can exert part of their regenerative effects through the release of extracellular vesicles (EV) or exosomes. EVs carry a wide range of bioactive cargo which includes nucleic acids, lipids, proteins, and a variety of intracellular mediators including cytokines. These can then fuse with other cells and incur transcriptional and translational modifications (77, 95) and facilitate intracellular communication (70). In a neonatal rat model of NEC, McCulloh et al. isolated EVs from four types of MSCs and injected them into the peritoneal cavity and found that EVs reduced the incidence of NEC in a dose-dependent manner and reduced histologic intestinal injury (83). No studies exist that specifically tie the use of MSCs and EVs in NEC and development of NDI, however, there are a few studies that look at the effects of EVs on perinatal brain injury.

A review of the therapeutic EV studies in experimental animal models of perinatal brain injury looked at 13 studies that administered EVs from MSCs *via* intravenous or intranasal administration in rats, mice, and sheep. The studies overall demonstrated an improvement in myelination and neuronal deficits following brain injury, decreased secretion of pro-inflammatory factors, and reduced microglia-mediated neuroinflammation (10). In rodent models of perinatal brain injury, long-term behavioral studies also demonstrated that EV treatment not only improved early neurological deficit scores, but improved long-term changes in motor coordination, spatial learning, and several types of memory testing (70). Thomi et al. looked at an *in vitro* model which showed that EV administration inhibited the production of pro-inflammatory cytokines by glial cells (including activated microglia) *via* interference of TLR4-signaling on microglial cells which prevented degradation of NFkB inhibitor and further downstream effects (10). In studies of HIE in preterm sheep, Ophelders et al. reported that intravenous administration of bone-marrow MSC-derived EVs to the fetus improved brain function (117). Collectively, these studies showed the benefits of EVs in improving neurodevelopmental outcomes, however, more studies with consistent cell lines and administration routes must be done to confirm these findings (70).

### 5.3. Nutritional supplementation

Probiotics are a group of supplements that have possible neuroprotective potential. They are an amalgam of micro-organisms that can help re-colonize the gut with commensal bacteria and improve gut barrier function. Many studies have shown a benefit in the risk of NEC, but little is known about the effects on NDI. Clinical studies by Alfalfa et al. and Akar et al. showed that probiotic supplementation in preterm and VLBW infants reduced the risk, incidence, severity, and all-cause mortality in NEC, however there was no clear effect of probiotics on neurodevelopmental outcomes (118, 119). In preclinical animal studies, probiotics have been shown ameliorate brain injury by releasing inhibitors of TNF-a and NF-*κ*B (36, 100, 101), blocking the transport of damaging bio-molecules *via* the GBA (36), alteration of mRNA expression in certain regions of the brain, and reducing HPA axis-induced release of cortisol (8, 120). In murine models, probiotics have been shown to alter anxiety and depression-related behavior in mice (8, 120) and in water-avoidance stress models strengthen tight junctional barrier integrity in the intestinal epithelium, which in turn attenuated the response of the HPA and ANS resulting in decreased end cortisol level and prevention of changes in the hippocampus (8, 121). Wang et al. showed that the probiotic *Lactobacillus reuteri* in a rodent model protected against several deleterious developmental behaviors such as cognition and anxiety and additionally prevented the increase in activated microglia and decrease in myelin basic protein that was seen in NEC (76).

Several studies have shown that early probiotic administration can help attenuate the effects of antibiotics and early life stress (9, 122–126), and prevent subsequent deleterious effects *via* the GBA. Cowan et al. performed studies that looked at maternal separation and early life stress in a rodent model. Pups in this model showed fear relapse and fear memories that more closely mimicked adult behavior (123). Female pups were shown to exhibit earlier onset of puberty while male pups exhibited an even later onset. Pups exposed to probiotics showed resistance to fear relapse and fear memories and restored normal onset of puberty in both sexes (124, 125). This maternal separation model also showed that by postnatal day 20, rats had hypercorticosteronemia, increased intestinal permeability, and altered gut microbiota—effects which were prevented in rats that were treated with probiotics. By postnatal day 56, rats exposed to maternal stress no longer showed serum changes in cortisol and their microbiome had largely normalized to control rats. However, the rats showed hypersensitivity when exposed to restraint stress with a significant increase in cortisol level and fecal frequency compared to controls. This hypersensitivity was also not seen in animals treated with probiotics (123). When applied to the pathophysiology of NEC and the GBA, probiotics could be a useful adjunct to attenuate the effects of early activations of brain-related circuits with fear and stress. However further studies need to be employed to look specifically at the effects of NEC and downstream NDI.

Prebiotics are defined as any substrate that is utilized by the host microbiota to confer a health benefit (9, 127). A main category is dietary fiber, which includes oligosaccharides, that may provide benefits to the developing preterm brain. These indigestible food components naturally occur in breast milk (human milk oligosaccharides) and have been assigned antimicrobial, immunomodulatory, and anti-inflammatory functions (127). Prebiotics have a high relative safety profile and can help the homeostasis of the gut microbiome (128). Fresh human milk provides up to a 4% relative risk reduction in the incidence of NEC (9) and helps to colonize the gut with healthy commensal bacteria and delivers important enzymes, immunomodulatory agents, and prebiotic oligosaccharides (36). Human milk contains many protective factors including secretory IgA, lactoferrin, and various oligosaccharides including glycosaminoglycans (GAGs). These carbohydrates are highly abundant and usually are not absorbed, but instead, serve as prebiotics for commensal bacteria in the intestine. They have been shown to exhibit immuno-modulatory effects in various disease processes (36). A prominent GAG gaining clinical interest in the treatment of NEC is chondroitin sulfate (CS), which comprises over half of the normal GAG content in human milk (129, 130) and is nonexistent in most major infant formulas (131). The concentration of CS in human breast milk is higher in preterm mothers than in term mothers indicating some evolutionary importance for preterm infants (44, 131, 132). In addition, maternal health characteristics have been shown to modulate the levels and function of GAGs, indicating that some element of maternal transfer is important to the health of infants (47) that are important to immune function and the development of a healthy microbiome (36). This in turn can prevent the deleterious activation of the GBA that can result in brain injury and downstream NDI (36).

Dabydeen et al. studied the effects of a high-calorie (120% of normal) and protein diet during the first year of life. With the altered diet, infants had dramatic improvements in head growth, weight gain, and increased axonal diameters in their corticospinal tracts. Unfortunately, neurodevelopmental data were unable to be obtained as the trial was aborted due to obvious benefits in the cohort with the diet. However, the importance of nutritional supplementation as an adjunct in the treatment of NEC and the potential for reducing NDI is important to continue to investigate (133). Taken together these studies demonstrate that nutrition, prebiotics, and probiotics can be important adjuncts to the treatment of NEC and amelioration of downstream NDI.

## 6. Conclusion

The morbidity and mortality of NEC in infants and the downstream neurodevelopmental complications after survival is well elucidated. Studies show that of infants that survive neonatal NEC, up to 45% of children show neurodevelopmental impairment (12). The pathophysiology of NEC involves a complex signaling cascade of the Gut-Brain axis driven by dysbiosis and inflammatory signaling within the intestine. This triggers inflammatory mediators that enter the systemic circulation, participate in TLR-4 signaling, or directly communicate between neural networks involving the ENS and the vagus nerve. Together these result in downstream microglial activation, subsequent astrocytic hypertrophy/astrogliosis, and impaired functioning of pre-oligodendrocytes, which ultimately cause white matter injury and impaired myelination (5–7, 70). This cascade inhibits normal brain development and growth, which is seen as white matter abnormalities on MRI (17, 25, 34). It becomes imperative therefore to make strides in therapies to protect against brain injury and downstream NDI. Although there is no clear therapeutic intervention to improve or prevent NDI, there is some promising early research in the field of stem cells, extracellular vesicles, probiotic/prebiotic therapies, and aggressive nutrition. With the prevalence and emotional burden that NDI following NEC carries on our society, it becomes important to continue research in this field—with a specific focus on understanding gut-brain signaling and possible mechanistic targets of therapeutic and preventative interventions.
